# Spin in the reporting, interpretation, and extrapolation of adverse effects of orthodontic interventions: protocol for a cross-sectional study of systematic reviews

**DOI:** 10.1186/s41073-019-0084-4

**Published:** 2019-12-19

**Authors:** Pauline A. J. Steegmans, Nicola Di Girolamo, Reint A. Meursinge Reynders

**Affiliations:** 1 Department of Orthodontics, Academisch Centrum Tandheelkunde Amsterdam (ACTA), University of Amsterdam, Gustav Mahlerlaan 3004, 1081 LA Amsterdam, The Netherlands; 2 Center for Veterinary Health Sciences, Oklahoma State University, 2065 W Farm Road, Stillwater, OK 74078 USA; 3 EBMVet, Via Sigismondo Trecchi 20, 26100 Cremona, CR Italy; 4 Department of Oral and Maxillofacial Surgery, Academic Medical Center, University of Amsterdam, Meibergdreef 9, 1105 AZ Amsterdam, The Netherlands; 5 Studio di Ortodonzia, Via Matteo Bandello 15, 20123 Milan, Italy

**Keywords:** Orthodontics, Reporting, Systematic review, Intervention, Spin, Misleading reporting, Misleading interpretation, Misleading extrapolation, Adverse effect, Adverse event, Harm, Safety

## Abstract

**Background:**

Titles and abstracts are the most read sections of biomedical papers. It is therefore important that abstracts transparently report both the beneficial and adverse effects of health care interventions and do not mislead the reader. Misleading reporting, interpretation, or extrapolation of study results is called “spin”. In this study, we will assess whether adverse effects of orthodontic interventions were reported or considered in the abstracts of both Cochrane and non-Cochrane reviews and whether spin was identified and what type of spin.

**Methods:**

Eligibility criteria were defined for the type of study designs, participants, interventions, outcomes, and settings. We will include systematic reviews of clinical orthodontic interventions published in the five leading orthodontic journals and in the Cochrane Database. Empty reviews will be excluded. We will manually search eligible reviews published between 1 August 2009 and 31 July 2019. Data collection forms were developed a priori. All study selection and data extraction procedures will be conducted by two reviewers independently. Our main outcomes will be the prevalence of reported or considered adverse effects of orthodontic interventions in the abstract of systematic reviews and the prevalence of “spin” related to these adverse effects. We will also record the prevalence of three subtypes of spin, i.e., misleading reporting, misleading interpretation, and misleading extrapolation-related spin. All statistics will be calculated for the following groups: (1) all journals individually, (2) all journals together, and (3) the five leading orthodontic journals and the Cochrane Database of Systematic Reviews separately. Generalized linear models will be developed to compare the various groups.

**Discussion:**

We expect that our results will raise the awareness of the importance of reporting and considering of adverse effects and the presence of the phenomenon of spin related to these effects in abstracts of systematic reviews of orthodontic interventions. This is important, because an incomplete and inadequate reporting, interpretation, or extrapolation of findings on adverse effects in abstracts of systematic reviews can mislead readers and could lead to inadequate clinical practice. Our findings could result in policy implications for making judgments about the acceptance for publication of systematic reviews of orthodontic interventions.

## Background

Readers of the biomedical literature mostly just screen the title and the abstract of an article without assessing the full publication [1]. The beneficial and adverse effects of interventions should therefore be transparently reported in these summaries and should not mislead its readers. Misleading reporting, interpretation, or extrapolation of study results is called “spin” [2–4]. We will assess in abstracts of both Cochrane and non-Cochrane reviews whether adverse effects of orthodontic interventions were reported or considered and whether spin was identified and what type of spin.

Titles and abstracts are the most read sections of biomedical papers [1], because assessing the full research article is often conditioned by paywalls or because of a lack of time or language issues of the readers [1]. Abstracts should therefore clearly and truthfully reflect the objectives, methods, results, and the interpretation of research findings. The standard for Methodological Expectations of Cochrane Intervention Reviews (MECIR) [5] has listed a series of highly desirable and mandatory items that should be consulted by reviewers when preparing the abstract of their reviews. Item R13 of the MECIR standard states that: “The Abstract of the review should aim to reflect a balanced summary of the benefits and harms of the intervention.” This mandatory item is particularly crucial for presenting adverse effects of health care interventions, because these effects are often poorly reported in systematic reviews [6]. Numerous epidemiological studies have also shown that the assessment and reporting of adverse effects of interventions in primary research studies is often suboptimal [7–11]. We adopted Cochrane’s definition of adverse effects: “An adverse event for which the causal relation between the intervention and the event is at least a reasonable possibility” [12, 13]. This definition and other key terminology in this manuscript are summarized in Table 1 [2–4, 12–15].

**Table 1:** Glossary of terms

Term	Definition
Systematic review	The Cochrane glossary [12] defines a systematic review as “‘A review of a clearly formulated question that uses systematic and explicit methods to identify, select, and critically appraise relevant research, and to collect and analyse data from the studies that are included in the review. Statistical methods (meta-analysis) may or may not be used to analyse and summarise the results of the included studies.”
Intervention review	Cochrane [14] defines an intervention review as follows: “Intervention reviews assess the benefits and harms of interventions used in healthcare and health policy.”
Orthodontic interventions	Steegmans et al. [15] define orthodontic interventions as follows: “Orthodontic interventions refer to the use of any type of orthodontic appliance that are used to move teeth or change the jaw size or position for orthodontic purposes. These interventions also include appliances to maintain or stabilize the results of orthodontic treatment, for example retainers.”
Adverse effect	Cochrane [12, 13] defines an adverse effect as “an adverse event for which the causal relation between the intervention and the event is at least a reasonable possibility.”
Spin [3]	“Distorted presentation of study results.”
Spin [3]	“A misrepresentation of study results, regardless of motive (intentionally or unintentionally) that overemphasizes the beneficial effects of the intervention and overstates safety compared with that shown by the results.”
Spin [2]	“A specific intentional or unintentional reporting that fails to faithfully reflect the nature and range of findings and that could affect the impression the results produce in readers.”
Misleading reporting related-spin [4]	“Incomplete reporting of the study results that could be misleading for the reader.”
Misleading interpretation related-spin [4]	Inadequate interpretation of the study results overestimating the beneficial effect of the intervention.
Misleading extrapolation related-spin [4]	Inappropriate generalization of the study results by inadequate (1) extrapolation from the population, interventions, or outcome actually assessed in the study to a larger population, different interventions, or outcomes, or (2) inadequate implications for clinical practice.
Spin (in the abstract) on adverse effects of interventions	Incomplete or inadequate reporting, interpretation, or extrapolation (or a combination of these variables) of findings on adverse effects of interventions in the abstract that could be misleading for the reader.

When presenting information on adverse effects in the abstract, it is also crucial that it does not mislead the reader. A distorted presentation of study results has been defined as “spin” [3], but more elaborate definitions are also used (Table 1). The term spin was first used in 1995 in the medical literature by Horton [16] and has been further subdivided into three categories [4]: misleading reporting-related spin, misleading interpretation-related spin, and misleading extrapolation-related spin (Table 1). Yavchitz et al. [17] have ranked the various types of spin according to their severity. The severest form of spin in abstracts of systematic reviews and meta-analyses was scored for “conclusions that contain recommendations for clinical practices that were not supported by findings” [17]. A high prevalence of the various types of spin has been identified in multiple epidemiological studies [4, 18–22]. Boutron et al. [18] found spin in 50% (36/72) of the conclusions sections of the main text of parallel-group RCTs and in 58.3% (42/72) of the conclusions sections of the abstracts. Spin was also common in diagnostic accuracy studies published in journals with high impact factors [22]. Lockyer et al. [21] showed that spin is a frequent phenomenon in abstracts of RCTs of wound treatments, and Lazarus et al. [4] identified at least one example of spin in 84% (107/128) of the abstracts of non-randomized intervention studies. Spin is in strong conflict with the Declaration of Helsinki [23] that states that: “Authors have a duty to make publicly available the results of their research on human subjects and are accountable for the completeness and accuracy of their reports.”

In this study, we will assess whether potential adverse effects of orthodontic interventions were reported or considered (i.e., discussed, weighed, etc.) in the abstract of systematic reviews. We will further assess whether spin was introduced regarding information on these adverse effects in the abstract, and we will categorize the types of spin (Table 1). We will assess these issues in the five leading orthodontic journals and those included in the Cochrane Database of Systematic Reviews. In these reviews, we will assess adverse effects such as pain as a result of tooth movement and the adverse effects defined by Preoteasa et al. (Table 2) [24]. Scoping searches in the orthodontic literature confirmed the knowledge gaps on our research questions. Our pilot studies quantified these gaps and confirmed the need to address these questions. We will assess these issues in systematic reviews, because they are increasingly consulted by patients [25] and when well-conducted systematic reviews are considered among the information sources with the highest level of evidence [26]. Our research questions are important, because incomplete or misleading information on adverse effects of interventions may have detrimental effects on the treatment of orthodontic patients.

**Table 2:** Adverse effects hypothetically linked to orthodontic interventions [24]

Subgroup	Description
Local adverse effects	
Dental	• Crown: decalcifications, decays, tooth wear, enamel cracks and fractures; discolorations, deterioration of prosthetic crown (as fracturing a ceramic one during debonding);
	• Root: root resorption, early closure of root apex, ankylosis;
	• Pulp: ischemia, pulpitis, necrosis;
Periodontal	• Gingivitis, periodontitis, gingival recession or hypertrophy, alveolar bone loss, dehiscences, fenestrations, interdental fold, dark triangles;
Temporomandibular joint	• Condylar resorption, temporomandibular dysfunction;
Soft tissues of the oral and maxillofacial region	• Trauma (e.g., long archwires, headgear related), mucosal ulcerations or hyperplasia, chemical burns (e.g., etching related), thermal injuries (e.g., overheated burs), stomatitis, clumsy handling of dental instruments;
Unsatisfactory treatment outcome	• Inadequate morpho-functional, aesthetic or functional final result, relapse, failure to complete treatment due to treatment dropout.
Systemic adverse effects	
Psychological	• Teasing, behavioral changes of patients and parents; discomfort associated with pain presence and aesthetic look discontents during orthodontic appliance usage;
Gastro-intestinal	• Accidental swallowing of small parts of the orthodontic device (tubes, brackets);
Allergies	• To nickel or latex;
Cardiac	• Infective endocarditis;
Chronic fatigue syndrome	
Cross infections	• From doctor to patient, patient to doctor, patient to patient.

Permission to reproduce this table was obtained on August 16, 2018, from InTech’s Publishing Ethics and Legal Affairs Department

## Objectives

The objectives of this research study are summarized in the following research questions:

Research questions

- In abstracts of systematic reviews of orthodontic interventions, were potential adverse effects of these interventions reported or considered (i.e., discussed, weighed, etc.)?
- In abstracts of systematic reviews of orthodontic interventions, was spin identified in the reporting, interpretation, and extrapolation of adverse effects?
- What was the prevalence of each type of spin?

## Methods

**Fig. 1:**
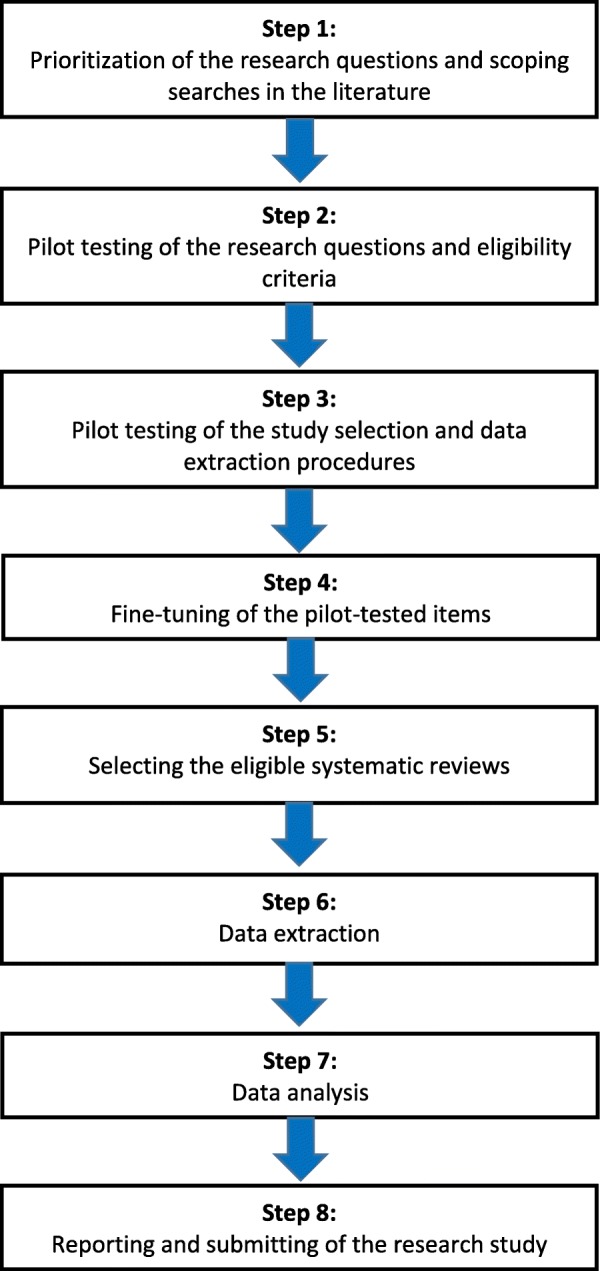
Flow diagram of the research methods. “Flow diagram of the research methods” was published previously by Steegmans et al. [15] in the journal “Systematic Reviews,” which is an open access journal of BioMed Central. Copyright on any open access article in a journal published by BioMed Central is retained by the author(s)

This protocol is reported according to the guidance of the Preferred Reporting Items for Systematic Review and Meta-Analysis Protocols (PRISMA-P) 2015 statement, and the PRISMA-P checklist is included as Additional file 1 [27, 28]. We adopted the same flow of research methods as reported in our published protocol on seeking adverse effects in systematic reviews of orthodontic interventions (Fig. 1) and conducted our pilot tests on the same sample of systematic reviews as was described in our previous protocol [15]. Our sample size of 14 reviews for the pilot test was calculated with the formula reported by Viechtbauer et al. [29]. Further details on the methods of our pilot test are reported in Additional file 2. This pilot test found that the reviewers in only 35.7% (5/14) of the abstracts reported or considered (i.e., discussed, weighed) potential adverse effects of orthodontic interventions. This sample identified an overall prevalence of 14.3% (2/14) of spin in the abstract on adverse effects of orthodontic interventions. Both cases of spin were “misleading reporting-related spin.” The following sections describe our planned methods based on these pilot tests. We will not start the selection of eligible reviews and data extraction prior to the complete acceptance of this protocol for publication.

### Eligibility criteria

We will adopt the same eligibility criteria that were defined for our published protocol on seeking adverse effects in systematic reviews of orthodontic interventions [15]. To avoid misinterpretation, we copied and pasted these eligibility criteria into Table 3 [15, 30].

**Table 3:** Eligibility criteria

Item	Included	Excluded
Study designs	Systematic reviews of orthodontic interventions. The definition of systematic review, intervention review, and orthodontic interventions listed in the Glossary of terms will be used to assess whether a review is eligible (Table 1).	(1) Non-interventional reviews such as, “Methodology,” “Diagnostic,” “Qualitative,” and “Prognostic” (2) Rapid and scoping reviews (3) Systematic reviews that focus exclusively on adverse effects of interventions (5) Systematic reviews of interventions that did not find any eligible studies (empty reviews)
Participants	Systematic reviews on any type of patients undergoing orthodontic interventions, i.e., patients of any health status, sex, age, demographics, and socio-economic status.	(1) Intervention reviews that focus exclusively on patients with congenital anomalies, for example with cleft lip and palate (2) Systematic reviews of animal or laboratory studies
Interventions	(1) Systematic reviews that assess the effects of clinical orthodontic interventions. Clinical orthodontic interventions refer to the use of any type of orthodontic appliance that is used to move teeth or change the jaw size or position for orthodontic purposes (2) Systematic reviews of interventions with appliances to maintain or stabilize the outcomes of orthodontic treatment, for example, retainers (3) Systematic reviews of orthodontic interventions that compare the effects of orthodontic treatment with or without additional interventions such as pharmacological or small surgical interventions, e.g., periodontal or implant surgery (4) No exclusion criteria will be applied to the characteristics of the operator who conducted the interventions	(1) Systematic reviews in which patients receive orthodontic treatment, but in which the effects of other interventions, e.g., periodontal surgery, are compared and not the effects of orthodontic interventions (2) Systematic reviews of interventions in which orthodontic appliances are specifically used for other purposes, e.g., changing jaw positions to treat respiration or temporomandibular disorders (3) Systematic review of orthodontic interventions that included orthognathic surgery
Outcomes	(1) Any adverse effect of orthodontic interventions scored at any endpoint or timing	No exclusion criteria
	(2) The effects of orthodontic interventions do not refer just to outcomes related to tooth and jaw size and positions, but also to broader outcomes such as periodontal health, esthetic changes, the health of the temporomandibular joint, patient health experiences, and economic issues associated with the interventions	
	(3) The reporting of outcomes on adverse effects will not determine the eligibility of reviews for this cross-sectional study, i.e., reviews will not be excluded because they did not provide “usable” data [30]	
Stetting	Any type of setting in which the interventions were conducted, i.e., university or private practice, etc.	No exclusion criteria

### Information sources

We will manually search the Cochrane library [14] and the websites of the five leading orthodontic journals to identify eligible systematic reviews published between 1 August 2009 and 31 July 2019. We chose this starting date because the first of August 2009 coincides with the launch of the Preferred Reporting Items for Systematic Reviews and Meta-Analyses (PRISMA) statement and its guidance paper on 21 July 2009 [27, 28]. Journal selection was based on two criteria: (1) the journal has been published for 10 years or more and (2) the impact factor. The journal citation reports by Clarivate Analytics were consulted to identify the five leading orthodontic journals based on impact factor [31]. The following five orthodontic journals fulfilled both criteria: European Journal of Orthodontics (EJO), American Journal of Orthodontics and Dentofacial Orthopedics (AJODO), Angle Orthodontist, The Korean Journal of Orthodontics, and Orthodontics and Craniofacial Research.

### Study records

#### Data management

- Two authors (PS and RMR) will conduct all study selection and data extraction procedures independently.
- Pilot tests were conducted to train both reviewers in applying these methods consistently and for calibration purposes [28].
- We will apply the following strategies in the case of disagreement between the two authors on the eligibility of a paper or the extraction of data: (1) discussions between reviewers, (2) rereading the paper, (3) or if necessary contacting its authors [32]. Persistent disagreements will be resolved through the consultation and arbitration of a methodologist (NDG).
- All eligible systematic reviews will be downloaded, and all extracted data will be collected in an Excel spreadsheet.

#### Selection process

- All titles and abstracts in the websites of the five orthodontic journals will be hand-searched to identify eligible reviews. The section “Dentistry and Oral health” will be searched in the Cochrane library for eligible Cochrane reviews [14].
- We will only include the latest version of a review when updates have been published.
- Authors will be contacted in the case of doubt regarding multiple publications of the same review. We plan to include the first publication, but will make this decision on a case by case basis and will report the rationale for this choice.
- Our selection procedures will be presented in a PRISMA flow diagram [32, 33].
- All included and excluded studies will be presented in tables, and the rationale for exclusion will be given for each excluded review.

#### Data collection process

- All eligible studies together with their supplemental files will be merged into binder PDFs, and pertinent search terms are linked to these documents to facilitate data extraction [34, 35].
- Eligible search terms were identified through searches in thesauri and in key articles on adverse effects [13, 36–40]. These terms are given in Additional file 3 and are identical to those used in our protocol on seeking adverse effects in systematic reviews of orthodontic interventions [15].
- Our pilot-tested data collection forms will be used for all data extraction procedures (Additional file 4). The PRISMA [32, 33] and the PRISMA-P [27, 28] checklists and guidance were consulted to develop these forms. The criteria for scoring pertinent data items are defined in these forms.
- The entire eligible review of both orthodontic and Cochrane reviews will be searched for data items, i.e., the text, tables, figures, and all supplemental files. The plain language summary in the eligible Cochrane reviews will not be searched for data items.
- When during the data extraction procedure changes are made in the data collection forms, we will present this with rationale in the section “Differences between the protocol and review.”

#### Scoring adverse effects of orthodontic interventions

- We will use the framework of categories of known orthodontic adverse effects as defined by Preoteasa et al. [24] (Table 2). We will also include pain as a result of tooth movement as an adverse effect. Potential adverse effects that are identified during data extraction will be discussed between the two reviewers (PS and RMR). We will report the rationale when including additional adverse effects and will categorize them.
- Ambiguous outcomes that could be interpreted as either a beneficial or an adverse outcome will not be scored as “adverse.” The rationale for this score will be given. We will only consider ambiguous outcomes as “adverse” when the review authors define these outcomes as such and make a strong case for this classification.

#### Scoring spin in the reporting, interpretation, and extrapolation of adverse effects of orthodontic interventions

- We will assess three types of spin, i.e., misleading reporting, misleading interpretation, and misleading extrapolation on adverse effects of orthodontic interventions in the abstract (Table 4). Each type of spin will be assessed separately for reviews that either did or did not seek adverse effects of interventions.
- To facilitate our scoring procedures and to reduce the risk of misinterpretation, we subdivided each type of spin into categories and defined each category (Table 4). We will score the presence of spin when spin is identified for one or more of these categories. The scoring procedures are summarized in Additional file 4. Pilot tests were conducted to assess the validity of these procedures.

**Table 4:** Types of spin in reviews that did or did not seek adverse effects of interventions

Definitions of the three types of spin	Reviews that sought adverse effects of interventions	Reviews that did not seek adverse effects of interventions
Misleading reporting (in the abstract) on adverse effects of interventions: “Incomplete or inadequate reporting in the abstract on the results of adverse effects compared with what is reported in the main text of the manuscript, which could be misleading for the reader.”	Categories: (1) Not reporting in the abstract on the results of the adverse effects that were reported in the main text of the review. (2) Selective reporting in the abstract on the results of the adverse effects that were reported in the main text of the review.	Categories: (1) Reporting on results of adverse effects in the abstract when adverse effects were not sought. (2) Reporting in the abstract that adverse effects were sought when they were not sought.
Misleading interpretation (in the abstract) on adverse effects of interventions: “Interpretation in the abstract on the results of adverse effects that is not consistent with what is reported in the main text of the manuscript and underestimates the adverse effects of the intervention.”	Categories: (1) Claiming in the abstract that the intervention is safe (has no or minimal adverse effects), despite concerning results on the adverse effects in the main text of the review, e.g., based on non-statistically significant results on adverse effects with wide confidence intervals [17]. (2) Downgrading in the abstract the importance of the adverse effects, despite concerning results on the adverse effects in the main text of the review. (3) Recommendations are made in the abstract for clinical practice that are not congruent with the concerning results on the adverse effects in the main text of the review [17].	Categories: (1) Claiming in the abstract that the intervention is safe (has no or minimal adverse effects) despite not having sought adverse effects. (2) Downgrading in the abstract the importance of the adverse effects, despite not having sought adverse effects. (3) Recommendations are made in the abstract for clinical practice despite not having sought adverse effects.
Misleading extrapolation (in the abstract) on adverse effects of interventions: “Overgeneralisation in the abstract of the study results to different populations, interventions, outcomes or settings than were assessed in the study despite evidence in the main text on concerning adverse effects on a different population, intervention, outcome or setting.”	Categories: (1) Results are extrapolated in the abstract to another population, intervention, outcome, or setting than were assessed in the review despite evidence in the main text on concerning adverse effects on a different population, intervention, outcome or setting.	Categories: (1) Results are extrapolated in the abstract to another population, intervention, outcome, or setting than were assessed in the review despite not having sought adverse effects.

### Outcomes and statistical analyses

**Fig. 2:**
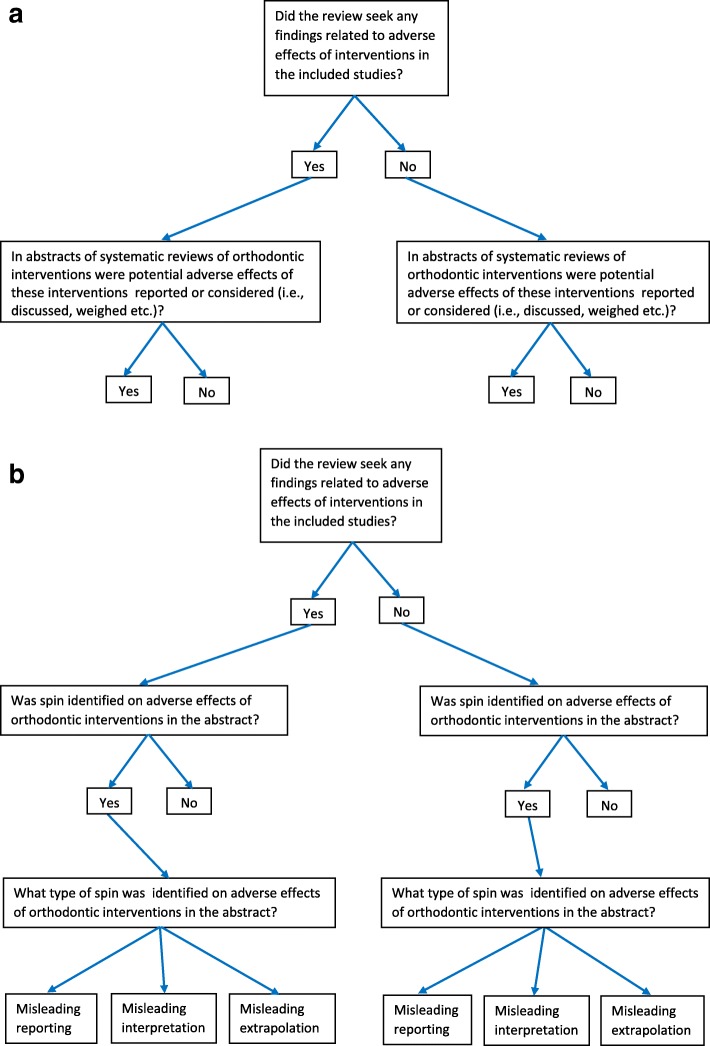
**a** Reporting or considering adverse effects of orthodontic interventions in the abstract. **b** Spin on adverse effects of orthodontics in the abstract

- Figure 2 a and b present all research questions in a flow diagram, and Table 5 lists all planned outcomes.
- We will calculate and report all prevalence data with their 95% confidence levels.
- We calculate the prevalence statistics for (1) all journals as one group, (2) the group of five leading orthodontic journals and the Cochrane Database of Systematic Reviews separately, and (3) each individual journal separately. Generalized linear models will be developed having the following outcomes for the abstracts of systematic reviews of orthodontic interventions: the reporting or considering of potential adverse effects of interventions/no reporting or considering of potential adverse effects of interventions (binary); presence of SPIN/absence of “SPIN” (binary); and misleading reporting/misleading interpretation/misleading extrapolation/no SPIN (categorical). The models will account for journal category (Cochrane Database of Systematic Reviews vs others), individual journals, and the geographical location of the study. Statistical significance will be based on a *p* value < 0.05. Stata software (Stata Corporation, College Station, TX, USA) version 15 will be used for all the statistical analyses [41].
- All outcomes that will be introduced or eliminated post hoc will be reported together with the rationale for inclusion or exclusion.

**Table 5:** Summary of findings

Description of outcomes from the main text	Statistic
The number of retrieved systematic reviews	Number
The number of eligible systematic reviews	Number
The prevalence of eligible systematic reviews	Prevalence
The prevalence of eligible systematic reviews that did seek any findings related to adverse effects of interventions in the included studies	Prevalence
The prevalence of eligible systematic reviews in which potential adverse effects of these interventions were reported or considered (i.e., discussed, weighed, etc.) in the abstract*	Prevalence
The prevalence of eligible systematic reviews in which spin was identified on adverse effects of orthodontic interventions in the abstract*	Prevalence
The prevalence of misleading reporting-related spin in the abstract*	Prevalence
The prevalence of misleading interpretation-related spin in the abstract*	Prevalence
The prevalence of misleading extrapolation-related spin in the abstract*	Prevalence

All prevalence data will be presented with their 95% confidence intervals

*This statistic will be reported for reviews that sought and did not seek any findings related to adverse effects of interventions in the included studies

### Reporting of the research study and data management

- The Strengthening the Reporting of Observational Studies in Epidemiology (STROBE) Statement will be used as the guideline for reporting the completed cross-sectional study [42].
- A data management plan was prepared for the long-term storage of our research data [43] in the case that the publisher of our completed research study will not or will only partly store our raw data. We consulted the Registry of Research Data Repositories [44] to identify an appropriate repository for our type of research data. We selected Dryad [45] for two reasons: (1) it is an international repository of data of peer-reviewed scientific and medical research and (2) it also includes data sets for which no specific data repository exist such as meta-epidemiological research data of systematic reviews in orthodontics. Our data management plan implies that (1) all our research data will be made freely available, (2) our completed article will present a link to a repository in which all raw data of the study will be deposited, (3) the repository is registered in the Registry of Research Data Repositories [44], (4) our research data will be reported in a format that permits other researchers to understand, cite, and reuse these data, (5) all sensitive data will be protected, and (6) it will be reassessed frequently and also updated if necessary [43, 44].

### Differences between the protocol and the completed study

- We will report all modifications between the protocol and the final research study. The rationale for each of these changes will be given.
- We will also report the consequences of these modifications on the magnitude, direction, and the validity of the outcomes [46].

## Discussion

### Strengths

Key strengths of this research study include the following: (1) we conducted extensive scoping searches and pilot studies to fine-tune our research questions and methods. These activities confirmed the importance of our questions. (2) Our research team consists of two topic experts (PS and RMR) and two methodologists (RMR and NDG). (3) All study selection and data collection procedures will be undertaken independently by two authors (PS and RMR). Calibration of these operators was done during the pilot studies. (4) To guarantee reproducibility and full access to our data, we will publish our protocol a priori and will include all raw data of the completed research study in additional files or will deposit them in an open-access repository [43–45, 47].

### Limitations

Including only orthodontic intervention reviews published in the five leading orthodontic journals and in the Cochrane Database of Systematic Reviews could be a limitation, but we expect that the findings in this subgroup of journals will underestimate the true severity of spin on adverse effects of interventions in the abstracts of these reviews. Including only reviews published in the last 10 years could also be a limitation. However, we chose this period because it brings the current knowledge status on our research questions to the foreground and these 10 years coincide with the launch in 2009 of the checklist of Preferred Reporting Items for Systematic Reviews and Meta-Analyses (PRISMA) [32, 33].

### Importance and beneficiaries

In this research study, we will address three key questions in abstracts of systematic reviews of orthodontic interventions: whether potential adverse effects of these interventions were reported or considered, whether spin was identified regarding information on these adverse effects, and the type of spin. These issues are important, because (1) the assessment and reporting of adverse effects of interventions is often suboptimal [7–11], (2) titles and abstracts are the most read sections of papers in the biomedical literature [1], (3) a high prevalence of spin has been identified in abstracts of both randomized and non-randomized studies [4, 21], and (4) incomplete or inadequate reporting, interpretation, or extrapolation of findings on adverse effects in the abstract can mislead readers and could lead to inadequate practice [4]. Our results will raise the awareness of considering adverse effects and the phenomenon of spin regarding these effects in abstracts of systematic reviews of orthodontic interventions. Patients, clinicians, researchers, editors, peer-reviewers, guideline developers, policy makers, and research funders will all be beneficiaries of the findings of this research study.

## Supplementary information

**Additional file 1.** Checklist for the Preferred Reporting Items for Systematic review and Meta-Analysis Protocols (PRISMA-P) 2015 statement.

**Additional file 2.** Pilot tests.

**Additional file 3.** Search terms and their derivatives.

**Additional file 4.** Data collection forms.

## Data Availability

Not applicable

## Abbreviations

MECIR: Methodological Expectations of Cochrane Intervention Reviews
PRISMA: Preferred reporting items for systematic reviews and meta-analyses
PRISMA-P: Preferred Reporting Items for Systematic review and Meta-Analysis-Protocols
