# Supplementary material for: Spin in the reporting, interpretation, and extrapolation of adverse effects of orthodontic interventions: protocol for a cross-sectional study of systematic reviews
Source: Res Integr Peer Rev. 2019 Dec 19;4:27. doi: 10.1186/s41073-019-0084-4 (PMC6921451; doi:10.1186/s41073-019-0084-4)
Supplement: Supplementary file 2 — Additional file 2. Pilot tests. [file 41073_2019_84_MOESM2_ESM.docx]

**Additional file 2. Pilot tests**

For our pilot studies we used the same sample of 14 reviews that was used for our pilot study of a previous protocol ‘Seeking adverse effects in systematic reviews of orthodontic interventions: protocol for a cross-sectional study’ [15]. The calculation of the sample size of this pilot study was based on the probability of the Yes scores for the question ‘‘Did the review seek any findings related to adverse effects of interventions in the included studies? This sample size was calculated using we the following equation [29]:

n = $\frac{ln(1-ϒ)}{\ln(1-\pi)}$

n = the sample size for the pilot study

ϒ = the threshold of confidence (95%)

π = the probability of a ‘Yes’ score

Our pilot test on our sample of 14 reviews found that reviewers in 35.7% (5/14) of the abstracts reported or considered (discussed, weighed etc.) potential adverse effects of orthodontic interventions. In this sample we identified a prevalence of 14.3 % (2/14) of spin in the abstract on adverse effects of orthodontic interventions. Both cases of spin were ‘Misleading reporting related spin’.
