# Supplementary material for: Spin in the reporting, interpretation, and extrapolation of adverse effects of orthodontic interventions: protocol for a cross-sectional study of systematic reviews
Source: Res Integr Peer Rev. 2019 Dec 19;4:27. doi: 10.1186/s41073-019-0084-4 (PMC6921451; doi:10.1186/s41073-019-0084-4)
Supplement: Supplementary file 4 — Additional file 4. Data collection forms. [file 41073_2019_84_MOESM4_ESM.docx]

**Additional file 4. Data collection forms**

**Table 1. Data collection form to identify eligible reviews**

| **Items** | **Description** |
| --- | --- |
| Journal | List the pertinent journal |
| Year | Year of publication |
| Binder page number | List the binder page number |
| Reference | List full reference (Authors, Title, Journal) |
| Is the article a systematic review? | Answer: Yes/No  Consider definition of a systematic review |
| What type of systematic review? | List the type of systematic review.  Consider different types of systematic reviews.  When the publication is not an intervention systematic review describe what type it is or could be and classify. Types of systematic reviews will receive a final classification during the discussions between operators. |
| Were orthodontic interventions assessed? | Answer: Yes/No  Consider the definition of orthodontic interventions. |
| What was the orthodontic intervention? | List the type of orthodontic intervention  NA: When the article is not a systematic review or not a systematic review of interventions. |
| Is the systematic review eligible? | Answer: Yes/No  Yes: The article is a systematic review of an orthodontic intervention.  No: The article is not a systematic review of an orthodontic intervention.  No: The article is a systematic review of an orthodontic intervention, but focusses exclusively on its adverse effects. |
| Page and potential comments* | Present the pertinent pages of reference for scoring the previous items and list the potential comments. |

*When referring to a particular page in the systematic review, we will use the page number of the systematic review and not the number in the binder document.

**Table 2. Data collection form on seeking any findings related to adverse effects of interventions in the included studies**

| **Items** | **Description** |
| --- | --- |
| Did the review seek any findings related to adverse effects of interventions in the included studies? | Answer: Yes/No  Yes: Any findings related to adverse effects of interventions in the included studies were sought by the reviewers.  Seeking any findings related to adverse effects of interventions in the included studies refers to reporting anywhere in the review (except in the Abstract) that such adverse effects in the included studies were sought.  Yes: Yes is also scored when reviewers only reported findings related to adverse effects of interventions in the included studies, but did not report that they actually sought them or planned to seek them. For example ‘Yes’ will be scored when outcomes on adverse effects of interventions in the included studies were reported in the review, but were not defined as objectives of the review.  Yes: Yes is also scored when the reviewers reported that they planned to seek (for example in the research objectives) findings related to adverse effects of interventions in the included studies, but did not report on these findings.  No: Findings related to adverse effects of interventions in the included studies were not sought by the reviewers. |
| In abstracts of systematic reviews of orthodontic interventions were potential adverse effects of these interventions reported or considered (i.e., discussed, weighed etc.)? | Answer: Yes/No  Yes: In abstracts of systematic reviews of orthodontic interventions potential adverse effects of these interventions were reported or considered (i.e., discussed, weighed etc.).  No: In abstracts of systematic reviews of orthodontic interventions potential adverse effects of these interventions were not reported or considered (i.e., discussed, weighed etc.). |

**Table 3a. Data collection form to identify spin in reviews that did seek adverse effects of interventions**

| **Items for misleading reporting (in the abstract) on adverse effects of interventions** | **Score** |
| --- | --- |
| 1) Not reporting in the abstract on the results of the adverse effects that were reported in the main text of the review. | Yes/no |
| 2) Selective reporting in the abstract on the results of the adverse effects that were reported in the main text of the review. | Yes/no |
| *Summary score on the presence of misleading reporting (in the abstract) on adverse effects of interventions* | *Yes/no*  *Yes is scored when one or more of the 2 items is answered with a ‘Yes’*  *No is scored when both items are answered with a ‘No’* |
|  |  |
| **Items for misleading interpretation (in the abstract) on adverse effects of interventions** |  |
| 1) Claiming in the abstract that the intervention is safe (has no or minimal adverse effects), despite concerning results on the adverse effects in the main text of the review, e.g., based on non-statistically significant results on adverse effects with wide confidence intervals [17]. | Yes/no |
| 2) Downgrading in the abstract the importance of the adverse effects, despite concerning results on the adverse effects in the main text of the review. | Yes/no |
| 3) Recommendations are made in the abstract for clinical practice that are not congruent with the concerning results on the adverse effects in the main text of the review [17]. | Yes/no |
| *Summary score on the presence of misleading interpretation (in the abstract) on adverse effects of interventions* | *Yes/no*  *Yes is scored when one or more of the 3 items is answered with a ‘Yes’*  *No is scored when all 3 items are answered with a ‘No’* |
|  |  |
| **Items for misleading extrapolation (in the abstract) on adverse effects of interventions** |  |
| 1) Results are extrapolated in the abstract to another population, intervention, outcome or setting than were assessed in the review despite evidence in the main text on concerning adverse effects on a different population, intervention, outcome or setting. | Yes/no |
| *Summary score on the presence of misleading extrapolation (in the abstract) on adverse effects of interventions* | *Yes/no*  *Yes is scored when the item is answered with a ‘Yes’*  *No is scored when the item is answered with a ‘No’* |

**Table 3b. Data collection form to identify spin in reviews that did not seek adverse effects of interventions**

| **Items for misleading reporting (in the abstract) on adverse effects of interventions** | **Score** |
| --- | --- |
| 1) Reporting on results of adverse effects in the abstract when adverse effects were not sought. | Yes/no |
| 2) Reporting in the abstract that adverse effects were sought when they were not sought. | Yes/no |
| *Summary score on the presence of misleading reporting (in the abstract) on adverse effects of interventions* | *Yes/no*  *Yes is scored when one or more of the 2 items is answered with a ‘Yes’*  *No is scored when both items are answered with a ‘No’* |
|  |  |
| **Items for misleading interpretation (in the abstract) on adverse effects of interventions** |  |
| 1) Claiming in the abstract that the intervention is safe (has no or minimal adverse effects) despite not having sought adverse effects. | Yes/no |
| 2) Downgrading in the abstract the importance of the adverse effects, despite not having sought adverse effects. | Yes/no |
| 3) Recommendations are made in the abstract for clinical practice despite not having sought adverse effects. | Yes/no |
| *Summary score on the presence of misleading interpretation (in the abstract) on adverse effects of interventions* | *Yes/no*  *Yes is scored when one or more of the 3 items is answered with a ‘Yes’*  *No is scored when all 3 items are answered with a ‘No’* |
|  |  |
| **Items for misleading extrapolation (in the abstract) on adverse effects of interventions** |  |
| 1) Results are extrapolated in the abstract to another population, intervention, outcome or setting than were assessed in the review despite not having sought adverse effects. | Yes/no |
| *Summary score on the presence of misleading extrapolation (in the abstract) on adverse effects of interventions* | *Yes/no*  *Yes is scored when the item is answered with a ‘Yes’*  *No is scored when the item is answered with a ‘No’* |
